# The composition of cell-based therapies obtained from point-of-care devices/systems which mechanically dissociate lipoaspirate: a scoping review of the literature

**DOI:** 10.1186/s40634-022-00537-0

**Published:** 2022-10-09

**Authors:** Perry Liu, Binay Gurung, Irrum Afzal, Matteo Santin, David H. Sochart, Richard E. Field, Deiary F. Kader, Vipin Asopa

**Affiliations:** 1 South West London Elective Orthopaedic Centre, Epsom, UK; 2 Centre for Regenerative Medicine and Devices, School of Applied Sciences, University of Brighton, Brighton, UK; 3 University of London, St George’s, London, UK

**Keywords:** Cell-based therapy, Stromal vascular fraction, Micro-fragmented fat, Nanofat, Mesenchymal stem cell, MSC, Adipose-derived stem cell, ASC, Osteoarthritis

## Abstract

**Purpose:**

Cell-based therapies using lipoaspirate are gaining popularity in orthopaedics due to their hypothesised regenerative potential. Several ‘point-of-care’ lipoaspirate-processing devices/systems have become available to isolate cells for therapeutic use, with published evidence reporting their clinical relevance. However, few studies have analysed the composition of their ‘minimally-manipulated’ cellular products in parallel, information that is vital to understand the mechanisms by which these therapies may be efficacious. This scoping review aimed to identify devices/systems using mechanical-only processing of lipoaspirate, the constituents of their cell-based therapies and where available, clinical outcomes.

**Methods:**

PRISMA extension for scoping reviews guidelines were followed. MEDLINE, Embase and PubMed databases were systematically searched to identify relevant articles until 21^st^ April 2022. Information relating to cellular composition and clinical outcomes for devices/systems was extracted. Further information was also obtained by individually searching the devices/systems in the PubMed database, Google search engine and contacting manufacturers.

**Results:**

2895 studies were screened and a total of 15 articles (11 = Level 5 evidence) fulfilled the inclusion criteria. 13 unique devices/systems were identified from included studies. All the studies reported cell concentration (cell number regardless of phenotype per millilitre of lipoaspirate) for their devices/systems (range 0.005–21 × 10^6^). Ten reported cell viability (the measure of live cells- range 60–98%), 11 performed immuno-phenotypic analysis of the cell-subtypes and four investigated clinical outcomes of their cellular products. Only two studies reported all four of these parameters.

**Conclusion:**

When focussing on cell concentration, cell viability and MSC immuno-phenotypic analysis alone, the most effective manual devices/systems were ones using filtration and cutting/mincing. However, it was unclear whether high performance in these categories would translate to improved clinical outcomes. Due to the lack of standardisation and heterogeneity of the data, it was also not possible to draw any reliable conclusions and determine the role of these devices/systems in clinical practice at present.

**Level of Evidence:**

Level V Therapeutic.

**Supplementary Information:**

The online version contains supplementary material available at 10.1186/s40634-022-00537-0.

## Introduction

The underlying principle of cell-based therapy is the targeted delivery of donor cells to achieve a medicinal benefit [28] and this has been long established in applications like bone marrow transplantation. There is now growing interest in orthopaedics as to whether cell-based therapies can be used to treat diseases such as osteoarthritis (OA), in the hope that they can repair damaged tissue and reduce the need for surgical intervention [43]. Mesenchymal stem cells (MSCs) are found in many locations around the body such as bone marrow and adipose tissue [23], with those from the latter termed adipose-derived stem cells (ASCs) [79].

Initially, it was believed that MSCs were the mediators of tissue repair because of their pluripotent ability to differentiate into cartilage and bone tissue [32]. However, due to an inability to control for differentiation in vivo, new evidence suggests that MSCs (when isolated) behave as pericytes and exert their regenerative effects through paracrine or immunogenic ways [13], rather than cell differentiation. It has therefore been suggested that the acronym ‘MSC’ be changed to ‘medicinal signalling cells’ accordingly [14].

**Fig. 1:**
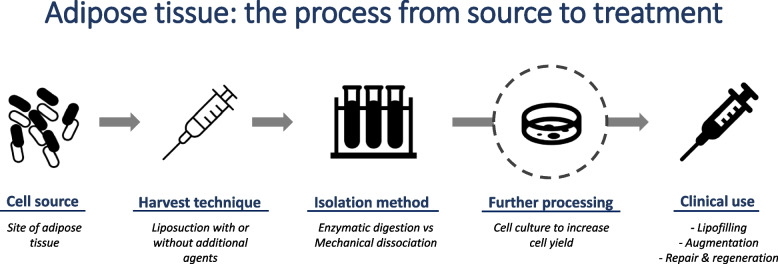
Schematic flowchart demonstrating the process of forming a cell-based therapy from adipose tissue

Small ASC numbers can be isolated in the cellular concoctions of mechanically dissociated and/or enzymatically digested lipoaspirate. Other cell-types present include fibroblasts, immune cells, epithelial cells and endothelial cells [11]. ASCs can be cultured to increase/expand their numbers [70], but this is time-consuming and unsuitable for point-of-care (POC) treatment [70]. Expansion also involves extensive cell manipulation, and it is unclear whether their properties can be preserved between culture and re-injection [5, 30, 53]. Therefore, using freshly processed lipoaspirate (containing heterogenous cells and not just ASCs) has become more popular [77] (Fig. 1). Although higher cell numbers are generated with enzyme digestion [4], these processes can alter cell architecture [60], so mechanical-only methods have now been favoured for this purpose.

These mechanical methods involve processes like centrifugation, filtration, cutting/mincing, decantation and washing. The inconvenience of needing various equipment at each stage has led to an increasing number of devices or systems that have been developed as ‘all-in-one’ options for easier therapeutic delivery [9]. Although studies have reported clinical benefit from using these devices/systems, little is known about the composition of their cell-based therapies and what is being reinjected into patients [4, 52]- information needed to help us understand how these therapies work. Therefore, the aim of this literature review was to summarise the available mechanical lipoaspirate-processing devices/systems and what they produce. Where available, the composition of their cellular products and clinical outcome data were compared in parallel.

## Methods

This study was in accordance with the Preferred Reporting Items for Systematic Review and Meta-Analysis extension for scoping reviews (PRISMA-ScR) guidelines [69] and was registered on the PROSPERO’s international prospective register of systematic reviews [CRD42021282041]. The five-stage scoping review process described by Arksey and O’Malley [3] was followed and adaptations from the Joanna Briggs Institute [48] were incorporated.

### Stage 1: Identifying the research question

#### A preliminary review of the literature showed that:

- 1) There was a paucity of information about these POC devices/systems.
- 2) Although clinical outcomes had been reported from using their cell-based therapies, it was unclear what was being reinjected into patients.

This led to the following research question being devised “What do these cell-based therapies contain?” (When using POC devices/systems which mechanically dissociate lipoaspirate).

### Stage 2: Identifying the relevant studies

MEDLINE and Embase databases were searched via the Healthcare Database Advanced Search (HDAS) engine from inception to date 1^st^ September 2021. A supplemental search of the native PubMed database was performed as well. A search syntax was formulated (Supplementary material- AdditionalFile1.docx) which focussed on four domains- cell type, adipose tissue, cell isolation and device/system.

Medical Subject Heading (MeSH)-terms and keywords were used to identify relevant articles. The searches were re-run on 21^st^ April 2022 in the Ovid search engine to capture any additional studies. All efforts were made to search the gray literature for relevant articles missed, including a manual search of the references of the included studies and relevant review articles.

### Stage 3: Study selection

After deduplication, two reviewers (PL, BG) independently screened the titles and abstracts for relevance. Following this, the full texts of the remaining articles were assessed for eligibility (Table 1). A third senior reviewer (VA) was consulted in the event of a disagreement about a study’s inclusion.

**Table 1:** Inclusion, exclusion and PICO (Population Intervention Comparison and Outcome) criteria for this review

Inclusion criteria		Exclusion criteria
-Published articles in English or with translation freely available (from any period) -Full text accessibility -Study designs (any of): Randomised control trials (RCTs), non-RCTs, prospective and retrospective cohort studies, cross-sectional studies, case–control studies, case series		-Adipose tissue obtained from animals or cadavers -Enzyme use prior to device/system administration or enzymatic-based devices/systems -Devices/systems involved in lipotransfer or the harvesting process of adipose tissue eg. AquaVage, and LipiVage [78] -Case reports, review articles, abstracts, letters and non-peer reviewed articles -Studies reported in any other language apart from English with no translation
**PICO criteria**		
**Population**		Human subjects (any age) including source of adipose tissue
**Intervention/Exposure**		Use of commercially available devices and systems to mechanically process lipoaspirate to obtain fresh cells
**Control/Comparator**		Any other lipoaspirate-processing technique
**Outcome(s)**	**Primary**	Cell concentration at point of isolation, without further expansion in culture
	**Secondary**	Cell viability, phenotypic analysis and clinical application of the cellular product

#### Outcome Measures (definitions):

- Cell concentration- Number of cells (irrespective of phenotype) per millilitre of processed lipoaspirate.
- Cell yield-Overall number of cells (irrespective of phenotype) that are present in the final product.
- Cell viability-A measure of the proportion of cells that are live and healthy [1].
- Cell phenotype- Hallmark characteristics of a cell and its surface markers.

To provide more information about the devices/systems captured in the included studies, an additional search of each device/system was performed in the PubMed database and Google search engine.

### Stage 4: Charting the data

Information about study characteristics (Table 2), laboratory analysis (Table 3) and immunophenotyping (Table 4) were extracted and tabulated in a database.

**Table 2:** List of included publications and their study characteristics

**Year of Study**	**Author**	**Affiliation with company of device/ system or other conflict**	**Study Design**	**Level of evidence (Therapeutic)**	**Journals**	**No. of donors for lipoaspirate**	**Clinical Outcomes measured?**
2015	Domenis et al. [24]	None	Prospective Cohort Study	2	Stem Cell Research and Therapy	6	Yes (Breast reconstruction)
2015	Gentile et al. [29]	None	Prospective Cohort Study	2	Plastic and Reconstructive Surgery	20 (10 for each device/system)	Yes (Breast reconstruction)
2016	Cicione et al. [16]	MyStem EVO kits donated by MyStem LCC	Descriptive laboratory study	5	Plastic and Reconstructive Surgery	14	No
2017	Dragoo et al. [27]	Adiprep kit donated by Harvest Technologies Corp	Descriptive laboratory study	5	The American Journal of Sports Medicine	7	No
2017	Morselli et al. [42]	None	Descriptive laboratory study	5	Wound Repair and Regeneration	18	No
2017	Streit et al. [63]	None	Descriptive laboratory study	5	Plastic and Reconstructive Surgery	14	No
2018	Tarallo et al. [65]	None	Prospective Cohort Study	2	Plastic and Reconstructive Surgery	20	Yes (Wound healing)
2018	Vezzani et al. [75]	Several authors have affiliations with Lipogems	Descriptive laboratory study	5	Stem Cells Translational Medicine	-	No
2019	Cohen et al. [17]	Several authors have affiliations with both Lipocube Ltd and Tulip Medical	Descriptive laboratory study	5	Aesthetic Surgery Journal	10	No
2019	Sese et al. [61]	Partially funded by Tulip Medical and kit donated by Tulip Medical	Descriptive laboratory study	5	Plastic and Reconstructive Surgery	6	No
2019	Winnier et al. [77]	Several authors hold positions at InGeneron, Inc	Descriptive laboratory study	5	Public Library of Science	12	No
2020	Copcu et al. [18]	None	Case Series	4	Aesthetic Surgery Journal	24	Yes (Fat grafting)
2020	Dai Pre et al. [20]	None	Descriptive laboratory study	5	International Journal of Molecular Sciences	9	No
2020	Tiryaki et al. [66]	Several authors have affiliations with both Lipocube Ltd and Tulip Medical	Descriptive laboratory study	5	Aesthetic Surgery Journal	10	No
2021	Busato et al. [12]	None	Descriptive laboratory study	5	Cells	27	No

**Table 3:** Summary of the mechanical devices/systems used in each study, their uncultured cell concentrations, viability (where applicable) and analytical techniques used

**Device/ System used *(Author)***	**Adipose donor site**	**Harvest technique and manipulation of lipoaspirate prior to insertion in device/ system**	**Volume processed (ml)**	**Cell Concentration (x10**^**6**^**/ml of lipoaspirate)**	**Cell Viability (%)**	**Estimated total cell yield of product (x10**^**6**^**)**^**a**^	**Laboratory analysis used to quantify cell numbers (after device/system processing)**						
			**Final volume of product (ml)**				**Enzyme use**	**Centrifugation**	**Filtration**	**Washing**	**Other Mechanical**	**Culture medium/ FBS/ Antibiotic**	**Counting Device**
Adinizer *(Copcu et al. * [18]*)*	Abdomen	Harvested with 2.8mm diameter cannula with tumescent solution and adrenaline. Predilution with saline in 50% of samples tested	5-20	1.22^b^	92.75^b^	1.13- 13.6 (Depending on volume used)		Y					LunaStem device
			1-12 (Variable)										
Adiprep *(Dragoo et al. * [27]*)*	Knee fat pad	Harvested during arthroscopy into AquaVage system. Then subjected to fractionisation and syringe emulsification.	30	0.486^c^	69.03^c^	0.99 (Mean)	Y	Y		Y		Y	Haemocytometer
			~2.95 (Mean)										
Fastem *(Domenis et al. * [24]*)*	Abdomen, hips and trochanter region	Harvesting procedure not mentioned. ‘Standardised procedural protocol’ not described.	No data	0.444 to 1^d^	-	N/A	Y	Y	Y				No data
Fastem and MyStem *(Gentile et al. * [29]*)*	No data	Harvesting procedure not mentioned.	80	0.03 and 0.005	98^e^	0.29 and 0.049		Y	Y			Y	Haemocytometer
			10										
Hy-Tissue SVF *(Busato et al. * [12]*)*	Abdomen	Harvested with 11G cannula with Klein tumescence solution, followed by decantation	25-30	0.041	-	N/A		Y				Y	CytoSMART counter
			No data										
Lipocube Nano & Tulip Nanotransfer *(Cohen et al. * [17]*)*	No data	Harvested with 2.4mm diameter cannula and then cleaned with Ringer’s lactate, sedimented and decanted.	10	2.24 and 1.44	96.05	N/A	Y	Y			Y		Muse Flow Cytometer
			No data (‘Pellet’ used)										
Lipocube SVF *(Tiryaki et al. * [66]*)*	Hip	Harvested with 3.5mm diameter cannula then decanted.	20	0.94	97.55	N/A					Y	Y	MuseCell Analyzer
			No data (‘Pellet’ used)										
Lipogems *(Vezzani et al. * [75]*)*	Abdomen	Harvested with 17G cannula either manually or vacuum assisted and mixed with saline	60	0.027	-	N/A	Y	Y	Y	Y		Y	Haemocytometer
			20-30										
Lull pgm *(Morselli et al. * [42]*)*	Abdomen	Harvesting procedure not mentioned. ‘Negative pressure’- not clarified.	30	2.4	-	N/A	Y	Y	Y		Y	Y	Cell Coulter counter
			10										
MyStem *(Cicione et al. * [16]*)*	No data	Harvested with MyStem 1.8mm blunt- tip cannula. Process not reported.	17-50	0.6	75.87	3.6- 10.7 (Depending on introduced volume)		Y					NucleoCounter
			8-23.5 (Variable)										
MyStem *(Tarallo et al. * [65]*)*	Abdomen	Harvested with local anaesthetic. ‘Standard protocol’- not described	30	0.83	74.3	0.62- 4.3	Y	Y				Y	NucleoCounter
			1-7 (Variable)										
Puregraft *(Streit et al. * [63]*)*	Abdomen	Harvested with 3.5mm diameter cannula with tumescent solution.	50	0.198	60	N/A	Y	Y		Y		Y	Haemocytometer
			No data (Pellet used)										
Rigenera *(Dai Pre et al. * [20]*)*	Thigh and Abdomen	Harvesting procedure not mentioned. Lipoaspirate mixed with equal volume of culture medium, FBS and antibiotics.	4	21	-	N/A		Y	Y			Y	Tryptan blue exclusion assay
			4										
Transpose RT *(Winnier et al. * [77]*)*	No data	Harvested with ‘standard procedure’- not described. Lipoaspirate mixed with lactated Ringer solution	25	0.084	61.7	0.16							NucleoCounter
			3										
Tulip Nanotransfer *(Sese et al. * [61]*)*	Abdomen	Harvested with Carraway Harvester cannula with tumescent fluid, then washed with saline.	20	6.63	76.8	50.9		Y				Y	NucleoCounter
			10										

^a^ Estimated total cell yield= Volume of product (ml) X Cell concentration (x10^6^/ml of lipoaspirate) X % Cell viability

^b^ Value given is an average obtained from the four different protocols used in the study

^c^ Figures from Layer 2 which resulted in the highest numbers

^d^ Enrichment performed in only 50% of lipoaspirate sample

^e^ Generalised figure for the study overall, not specific to either device/system

**Table 4:** Immuno-phenotypic analysis performed and CD Marker Expression

**Device/ System used** ***(Author)***	**Type of immuno-phenotypic analysis of cell subtypes**	**Terminology for uncultured, freshly isolated cells**	**Stage of cell processing**	**Positive cell CD marker expression (%)**											
				Mesenchymal stem cell markers *CD markers observed in Pericytes as well							**Endothelial cell, pericyte and haematopoetic markers**				
				**CD 13**	**CD 29**	CD 44*	**CD 73**	CD 90*	CD 105*	CD 146*	**CD 31**	**CD 34**	**CD 45**	**CD 68**	**Other**
Adinizer *(Copcu et al. * [18]*)*	Flow Cytometry	Stromal cells/ Nuclear cells	Immediately after device use (minimally manipulated)	As per methods- Proportions of CD45 negative cells were analysed in CD34−CD146+ and CD34+CD146−CD90+ (deemed as regenerative perivascular cells), and CD34+CD146+ as endothelial cells. However, percentages not specifically reported in results.											
			No control												
			Passage in culture following device (extensively manipulated)												
Adiprep *(Dragoo et al. * [27]*)*	Flow Cytometry	SVF Cells	Immediately after device use (minimally manipulated)	56.5		72.0	60.4	65.2	33.4				80.3		
			No control												
			Passage in culture following device (extensively manipulated)	94.3		96.6	97.0								
Fastem *(Domenis et al. * [24]*)*	Flow Cytometry	SVF Cells	Immediately after device use (minimally manipulated)										50-60		**CD34+CD45-CD31-** 10-20
			Control- ‘modified’ Coleman’s procedure (centrifugation)										0-10		**CD34+CD45-CD31-** 20-30
			Passage in culture following device (extensively manipulated)												
Fastem and MyStem *(Gentile et al. * [29]*)*	Not done	SVF Nucleated Cells													
Hy-Tissue SVF *(Busato et al. * [12]*)*	Flow Cytometry	Free nucleated SVF cells	Immediately after device use (minimally manipulated)				7.61		6.28	2.6		9.91	5.5	3.5	**CD116** 0.7
			Control- enzymatic digestion using 0.1% collagenase type I at 37 °C for 45min followed by centrifugation at 400G for 10min.				10.1		9.98			3.67			
			Passage in culture following device (extensively manipulated)		90	>90	>70	60	90						
Lipocube Nano *(Cohen et al. * [17]*)*	Flow Cytometry	SVF Cells	Immediately after device use (minimally manipulated)	42.0			53	55.8		53.2		18.8			
			No control												
			Passage in culture following device (extensively manipulated)												
Lipocube SVF *(Tiryaki et al. * [66]*)*	Flow Cytometry	Nucleated SVF Cells	Immediately after device use (minimally manipulated)			21.5	6.16	11.4	9.0						
			Control- enzymatic digestion using GMP grade collagenase NB6 at a concentration of 0.1 U/ml at 37 °C for 30min followed by centrifugation at 400G for 10min. Then washed with PBS solution and centrifuged at 300G for 5min.			6.93	3.44	5.88	3.06						
			Passage in culture following device (extensively manipulated)												
Lipogems *(Vezzani et al. * [75]*)*	Flow Cytometry	SVF Nucleated Cells	Immediately after device use (minimally manipulated)												**CD146+CD34** 33.5 **CD34+CD146** 5.46
			No control												**CD146+CD34** 8.39 **CD34+CD146** 51.5
			Passage in culture following device (extensively manipulated)	CD14, CD31, CD40 ligand (CD154) significantly more abundant than when compared to control.											
Lull pgm *(Morselli et al. * [42]*)*	Not done	SVF Cells													
MyStem *(Cicione et al. * [16]*)*	Flow Cytometry	Lipoaspirate fluid cells	Immediately after device use (minimally manipulated)			<0.1		1-1.5	<0.1		0.5-1	<0.5	<1		
			Control- centrifugation ‘as previously described’			<0.1		1.5-2	<0.1		1	<0.5	<0.5		
			Passage in culture following device (extensively manipulated)			93	98	95	96						
MyStem *(Tarallo et al. * [65]*)*	Flow Cytometry	Freshly isolated LAF Cells	Immediately after device use (minimally manipulated)				0-10	75	0-10			0-10	20		**CD31** 30
			No control												
			Passage in culture following device (extensively manipulated)	All culture-expanded cells displayed an ASC-like immunophenotype: CD105+, CD73+, CD90+, CD45- and CD34-CD31.											
Puregraft *(Streit et al. * [63]*)*	Direct Immunofluorescence	SVF Cells	Immediately after device use (minimally manipulated)	Analysed adhesive properties to determine stem cell nature. All adherent cells were positive for CD90 and CD105 and negative for CD31 and CD45 antigens (stem cell marker). Numbers not specified.											
			Control 1- aliquot was left at 37°C for 20min under the action of gravity (decantation). Control 2- aliquot centrifuged at 1200G for 3 min.												
			Passage in culture following device (extensively manipulated)												
Rigenera *(Dai Pre et al. * [20]*)*	Flow Cytometry	Total cells	Immediately after device use (minimally manipulated)									3.12	4.98		**CD44/CD90** 30.4 **CD73/CD105** 16.6 **CD73/29** 27.8
			Control- enzymatic digestion using 0.1% collagenase type I at 37 °C for 45min in Hank’s Balanced Salt Solution (HBSS) and 2% bovine serum albumin followed by centrifugation at 3000 rpm for 7min.									76.7	7.32		**CD44/CD90** 48.1 **CD73/CD105** 54.3 **CD73/29** 62
			Passage in culture following device (extensively manipulated)	Expression of the typical mesenchymal stem cell markers (CD105, CD90, CD73, CD44, and CD29) and the hematopoietic markers (CD45 and CD34) was preserved through culture passages.											
Transpose RT *(Winnier et al. * [77]*)*	Not done	Adipose-derived regenerative cells													
Tulip Nanotransfer *(Cohen et al. * [17]*)*	Flow Cytometry	SVF Cells	Immediately after device use (minimally manipulated)	18.3			50	42.1		24.1		7.9			
			No control												
			Passage in culture following device (extensively manipulated)												
Tulip Nanotransfer *(Sese et al. * [61]*)*	Not done	Nanofat cells													

The separate search of each device/system was used to ascertain their individual characteristics and use in clinical applications (Table 5). The manufacturer website for each was also analysed for relevant information and peer-reviewed literature. Where possible, companies were contacted by email for any additional articles.

**Table 5:** Device/system characteristics and clinical applications in literature

**Device/ System**	**Company and location**	**Level of automation**	**Processing Time (mins)**	**Mechanical techniques used by device/system**						**Clinical Applications in PubMed indexed studies**
				Centrifugation	Filtration	Cutting / Mincing	Sedimentation/ Decantation	Washing	Other (Specify)	
Adinizer	BSLrest, Busan, South Korea	Manual	Variable (operator dependent)			Y				**Indication:** Fat grafting/ Lipofilling **Treatment ** [18]**: **Cellular product applied at varying depths to different aesthetic units of the face in 24 patients. **Outcome:** Visual analog scale (VAS) scores at 2 years were consistently high (Range 6-9) from both patient and surgeon.
Adiprep system (+ Smartprep)	Harvest Technologies Corp. Plymouth, MA, USA	Manual + Automated	4	Y					Emulsification	None
Fastem	CORIOS Soc. Coop, Milan, Italy	Automated	10	Y	Y					**Indication:** Fat grafting **Treatment ** [24]**: **Cellular product used to enrich fat grafts before breast augmentation in six patients, comparing their clinical results with patients who underwent grafting with standard lipoaspirate (n=16). **Outcome:** Greater gain of thickness of both the central and superior-medial quadrants at 6 months vs control.
Hy- Tissue SVF	Fidia Farmaceutici S.p.A, Padua, Italy	Manual	15		Y		Y		Massage	**Indication:** Osteoarthritis (Animal in vitro study) [22] **Indication: **Achilles tendinopathy **Treatment ** [71]: 21 patients with non-insertional achilles tendinopathy (28 tendons) were treated unilaterally or bilaterally with autologous cellular product. **Outcome: **Significant improvements in VAS, AOFAS and VISA-A scores at 15 and 30 day follow up intervals vs PRP group.
Lipocube Nano	Lipocube Inc, London, UK	Manual	20-30		Y	Y			Emulsification	None
Lipocube SVF/ CellDrive	Lipocube Inc, London, UK	Manual + Automated	20-30	Y	Y	Y				**Indication:** Fat grafting **Treatment ** [67]**: **SVF cell-enriched fat grafting in 46 patients for various aesthetic and reconstructive applications. **Outcome: **No complications. Results on a 4-point patient satisfaction scale ranged from good to excellent.
Lipogems	Lipogems International S.p.A, Milan, Italy	Semi- automated	3-5		Y		Y	Y	Shaking, Emulsification	**ENT ** **Indication:** Vocal cord palsy **Treatment ** [54]**:** 3 patients had laryngoplasty and injection of autologous cellular product. **Outcome:** At 12-month follow-up period, voice improvement was consistent in all patients. **General Surgery** **Indication: **Intersphincteric anal lipofilling **Treatment ** [15]: 3 patients with faecal incontinence had autologous cellular product injected in the intersphincteric anal groove. **Outcome:** At 1 month post procedure, each patient had an improved Wexner incontinence score. At 6 months, ano-rectal manometry showed an increase of resting pressure and ultrasound showed increased thickness of the sphincter. **Indication: **Repair of a vesicouterine fistula **Treatment ** [62]: 1 patient had endoscopic transurethral resection of the fistulous tract and injection of autologous cellular product. **Outcome:** 3 months post procedure, patient was asymptomatic. Cystoscopy showed appropriate scar tissue and cystogram revealed complete repair of VUF. At 24 months, there were no recurrences. **Orthopaedic Surgery** **Indication: **Osteoarthritis **Treatment ** [7]: 20 patients with knee OA were injected with autologous cellular product and followed up at various intervals. **Outcome: **Improvements in Knee injury and Osteoarthritis Outcome Score (KOOS) were significant at 3-,6- and 12-months follow-up. At one year, there were improvements in KOOS pain= 14 points, symptoms= 7, activities of daily living= 13, sports= 19 and quality of life=15. **Treatment ** [76]: 25 patients with shoulder OA were injected with autologous cellular product and followed up at various intervals. **Outcome: **At one-year, significant improvement (p<0.001) in Visual Analog Scale and disabilities of the arm. **Treatment ** [21]: 6 consecutive patients with hip OA were given single intra-articular injection of autologous cellular product and followed up at 6 months. **Outcome: **Harris Hip Score improved from 67.2 (mean pre-operative value) to 84.6 (mean pre- post-operative value) **Treatment ** [47]: 17 patients with knee OA treated with ultrasound-guided intra-articular injection of autologous cellular product and followed up for up to 12 months. **Outcome: **Knee Society Score improved from average 74 (baseline) to 82 (12 months) **Treatment ** [59]: 20 patients with temporomandibular OA treated with autologous cellular product after arthrocentesis vs control group (hyaluronic acid instead). Follow up for up to 6 months. **Outcome**: Treatment group had a statistically significant superiority in the success rate compared with the control group (P = .018). **Treatment ** [72]: 64 patients with symptomatic mild-severe knee OA treated with autologous cellular product. Follow up for up to 12 months. **Outcome**: KOOS, NRS and EQ-5D improved significantly at follow-up compared to baseline (p < 0.05). **Treatment ** [57]: 52 patients with early knee OA treated with autologous cellular product after arthroscopic debridement. Follow up for up to 24 months. **Outcome**: The IKS function score improved from average 57.2 (pre-operatively) to 83.0 (at the latest follow-up) (p<0.01). **Treatment ** [73]: 23 patients with early to moderate patellofemoral OA treated with autologous cellular product. Mean follow-up was 22.1 months. **Outcome**: Significant improvements in mean IKS knee and function scores vs baseline (35.6 to 61.9 and 52.0 to 82.3 respectively). **Treatment ** [58]**:** 202 patients with OA (Kellgren-Lawrence I-IV) were injected with autologous cellular product. Mean follow-up was 24.5 months. **Outcome:** At 6 months, Total KOOS significantly improved from baseline (p 0.001) and between 6-12 months. At 6 months, VAS was reduced vs baseline (p 0.001), increased at 12 months but remained below baseline. **Indication: **Used with High Tibial Osteotomy (HTO) for correction of varus knee OA **Treatment ** [36]: 42 patients treated with HTO and simultaneous intra articular injection of cellular product vs 43 patients treated with only HTO. **Outcome: **No significant results between both treatment groups in terms of KOOS pain, symptoms, sports, and quality of life. However, a significant improvement (p<0.05) in the activities of daily living.

### Stage 5: Collating, summarising, and reporting the results

Due to heterogeneity of the data, a formal meta-analysis could not be performed. A narrative analysis of the POC devices/systems, the composition of their therapies, and clinical outcomes (where available) was conducted.

The Oxford Centre for Evidence-Based Medicine (OCEBM) checklist [80] for therapeutic studies was used to assess the level of evidence of the included studies. Quality review of the studies was performed using a modified ‘Minimum Information for Studies Evaluating Biologics in Orthopaedics (MIBO)’ checklist presented by Murray et al. [45], which has been designed specifically for MSC-related studies. Adaptations from the STROBE assessment tool [19] were incorporated for assessing study design. A ‘heat map’ of reporting was subsequently generated (Fig. 3). The tool was validated by the same two reviewers (PL and BG) independently analysing the various domains.

## Results

### Search results

**Fig. 2:**
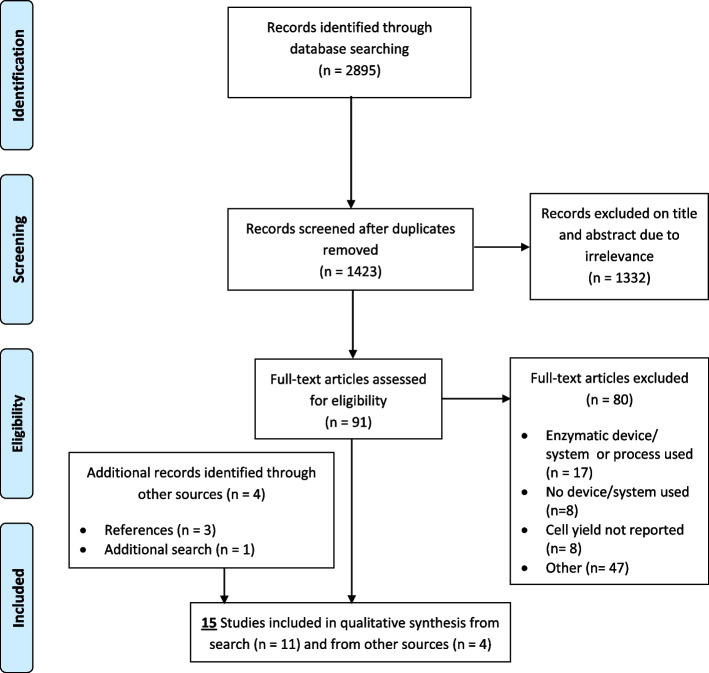
PRISMA flow diagram for search results (adapted from Moher et al [40])

From the primary search 11 studies fulfilled the inclusion criteria. Four additional studies were identified through other means (*n* = 3 through references, *n* = 1 additional search), leaving a total of 15 studies for qualitative synthesis (Fig. 2) [40]. Emailing the manufacturers for additional information resulted in five responses (BSLrest- Adinizer, Harvest Technologies Corp- Adiprep + SmartPrep, Tulip Medical- Tulip Nanotransfer, Cytori Therapeutics- Puregraft and Fidia Farmaceutici S.p.A- Hy-Tissue SVF). No new articles for inclusion were identified by these means, but some were used to populate Table 5.

### Level of evidence

Most of the included studies were low level evidence (Table 2) [12, 16–18, 20, 24, 27, 29, 42, 61, 63, 65, 66, 75, 77] 11 were Level 5 [12, 16, 17, 20, 27, 42, 61, 63, 66, 75, 77] (descriptive laboratory studies), one was Level 4 [18] and only three were Level 2 [24, 29, 65].

### Quality Assessment (Fig. 3)

**Fig. 3:**
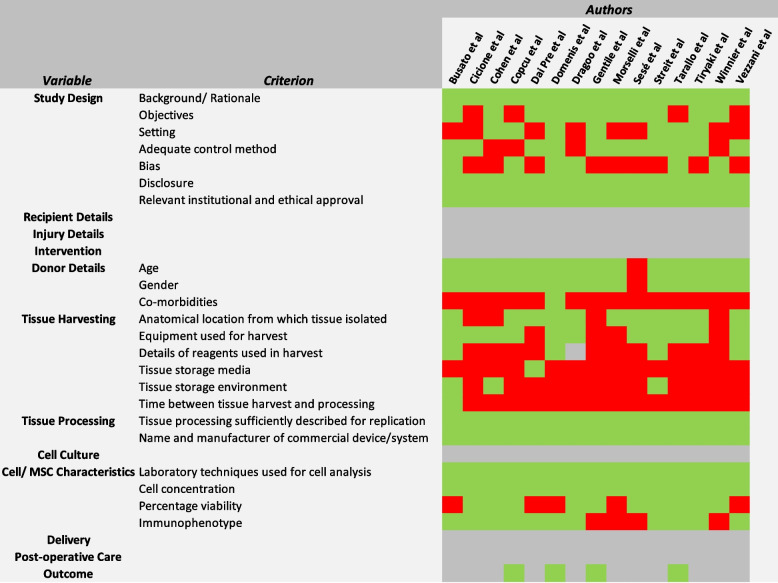
Modified MIBO checklist for the assessment of methodological quality of included studies, with adaptations from the STROBE assessment tool: Heat map of reporting (Green- Adequate reporting of variables, Red- Inadequate or unreported, Grey- Variables not applicable to individual studies)

All included studies [12, 16–18, 20, 24, 27, 29, 42, 61, 63, 65, 66, 75, 77] disclosed whether they had any financial or other competing interests. 73.3% (*n* = 11/15 [12, 17, 20, 24, 27, 29, 42, 61, 63, 66, 77]) gave a clear objective which reduced the risk of outcome bias. 26.6% (*n* = 4/15 [17, 18, 27, 77]) lacked an adequate control group which may have resulted in interpretation bias or publication bias. Most red fields in the heat map were for the ‘Donor details’ and ‘Tissue harvesting’ domains. Notably, only one study [24] reported donor co-morbidities, one [20] reported the media for tissue storage following harvest, and one [12] the time between tissue harvest and processing.

## Cell concentrations

All studies reported a concentration for freshly isolated cells following harvest and device/system administration (Table 3). There were varying definitions for these heterogenous minimally manipulated cells, the most common term that was used was ‘SVF cells’ (*n* = 9) (Table 4).

Dai Pre et al. [20] reported the highest concentration achievable (21 ± 0.16 × 10^6^per ml/ lipoaspirate) using the device/system Rigenera. For all devices/systems, mean concentration was 2.30 × 10^6^/ml overall ± 4.92 × 10^6^ (standard deviation). The next highest concentrations were Sese et al. [61] (6.63 ± 0.47 × 10^6^/ml- Tulip Nanotransfer), Morselli et al. [42] (2.4 × 10^6^/ml- Lull pgm) and Cohen et al. [17] (2.24 × 10^6^/ml and 1.44 × 10^6^/ml- Lipocube Nano & Tulip Nanotransfer) accordingly.

### Cell viability

Only two thirds of the studies (*n* = 10) [16–18, 27, 29, 61, 63, 65, 66, 77] gave a cellular viability in conjunction with their concentration (Table 3), the highest being Gentile et al. [29] with 98% using Fastem and Mystem. However, this viability figure was quoted for both devices overall rather than a specific one for each of the device’s products. The next highest figure was 97.55% for Tiryaki et al. [66] using Lipocube SVF.

**Fig. 4:**
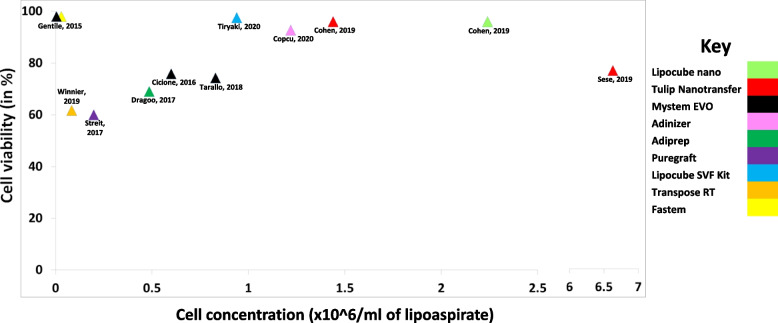
Scatter graph of studies and their reported cell concentrations and viability (Studies without viability figures were omitted)

For devices/systems with an associated viability figure, mean viability was 80.2% ± 14.0% (standard deviation). The study with the highest cell number with a viability over 90% was Cohen et al. [17] using Lipocube Nano and Tulip Nanotransfer (Fig. 4).

### Immuno-phenotypic analysis

Ten studies [12, 16–18, 20, 24, 27, 65, 66, 75] used flow cytometry analysis to immuno-phenotype the cell subtypes, whereas one [63] opted for direct immunofluorescence (Table 4). Positive mesenchymal stem cell markers of CD73, CD90 and CD105 (as specified by the ISCT- International Society for Cellular Therapy [25]), as well as CD44 and CD146 (also found in pericytes [6]) were reported at varying degrees across all studies. Six studies [12, 16, 17, 27, 65, 66] reported percentages for at least one of these markers in their population of cells following device/system use.

The devices/systems with the highest percentages of MSC CD markers following minimal manipulation were Adiprep- Dragoo et al. [27] (CD73 60.4%, CD90 65.2%, CD105 33.4%), Lipocube Nano- Cohen et al. [17] (CD73 53%, CD90 55.8%) and Tulip Nanotransfer- Cohen et al. [17] (CD73 50%, CD90 42.1%).

Six studies [12, 16, 20, 24, 63, 66] performed immunophenotypic analysis on a control method as well (either enzymatic or mechanical); two [24, 63] for mechanical, with a large difference only observed with Fastem [24]. Three studies [12, 16, 27] performed analysis of the MSC phenotype following culture and consistently achieved above 90% for CD markers 73,90,105.

### Devices/systems and their individual characteristics

Out of the 15 studies, 13 unique mechanical devices and systems were identified (Table 5). Five were manufactured by companies in the USA and four in Italy. Traditionally, the mechanical processes used have been centred around three main techniques: decantation, centrifugation and filtration [8]. More novel methods have now been introduced including the physical disruption of tissue, washing and cutting. The most popular techniques adopted were filtration (*n* = 10), washing (*n* = 5) and cutting/ mincing (*n* = 5).

### Clinical applications

Only four of the included studies [18, 24, 29, 65] assessed clinical outcomes following the use of their device/systems (Table 2). Copcu [18], Domenis [24] and Gentile [29] reported positive outcomes following contouring procedures. Tarallo [65] reported wound healing improvement using MyStem EVO.

Other clinical applications have been highlighted in Table 5 [7, 15, 21, 22, 33–39, 47, 51, 54, 55, 57–59, 62, 67, 71–73, 76]. None of the authors reported the constituents of the cellular therapies used in these studies. Lipogems [7, 21, 36, 47, 57–59, 72, 73, 76], MyStem EVO [55] and Hy-Tissue SVF [71] were the only device/systems to have been used in orthopaedic application.

## Discussion

This scoping review identified 13 unique mechanical devices/systems from 15 articles that fulfilled the inclusion criteria. The mean cell concentration (cell number generated per millilitre of processed lipoaspirate) from these devices/systems was 2.30 × 10^6^/ml of lipoaspirate (Table 3). Ten of 15 studies gave a cellular viability in conjunction with their concentration (mean 80.2%). 11 studies performed immuno-phenotypic analysis to characterise cell-types (Table 4), with six reporting markers for MSCs. Four studies assessed clinical outcomes. Only two studies [18, 65] reported all four parameters.

The mean cell concentration (2.30 × 10^6^/ml) was higher than concentrations obtained by conventional mechanical methods not using a POC device/system, as shown by Aronowitz et al. [4] (0.01–0.24 × 10^6^). It is possible that concentrations are greater following device/system use because of reduced handling and processing times. Nonetheless, this figure was skewed by one study [20] which did not report cell viability.

Viability is the proportion of live and metabolically active cells in the sample, so POC devices/systems should aspire for a cell viability as close to 100% as possible. The International Federation for Adipose Therapeutics and Science (IFATS) has since proposed a minimum threshold of 70% [10] for cells, but this was to allow for good cell expansion. Only nine devices/systems (seven studies) reported a cell viability above 70% [16–18, 29, 61, 65, 66]. Of these, the mean cell concentration was 1.55 × 10^6^ (0.005–6.63 × 10^6^). This was still higher than that of previously published literature [4], which indicates the therapeutic promise that these POC devices/systems may present.

However, this places significant weight on cell concentration as a variable. The cell yield (total number of cells delivered to the patient) is affected by the volume of the final product, as well as cell concentration. This varies across studies (Table 3) and depends on the therapeutic indication that is required. Additionally, evidence for a correlation between cell number and observed clinical benefit is inconclusive at present [50]. Theoretically, higher cell concentrations should result in higher ASC numbers (when accounting for the final volume of product) and therefore better outcomes, but this hypothesis is making the assumption that ASCs are the critical cell type in achieving clinical benefit. If so, the most effective devices/systems were the Tulip Nanotransfer which isolated 6.63 × 10^6^ cells/ml at 76.8% viability and Lipocube Nano- 2.24 × 10^6^ cells/ml at 96.05% viability; the highest concentrations and viability combined (Fig. 3). These devices/systems utilise filtration and cutting/mincing in their processing, and avoid other steps such as centrifugation, sedimentation and washing, hence the terms microfragmented adipose tissue (MFAT) or nanofat [31] being used in the literature to describe the processed lipoaspirate.

On the other hand, there was variability in the concentrations obtained from these devices/systems [17, 61] and others across different studies. Therefore, it is unclear whether the higher concentrations obtained overall were significant or erroneous. It is likely that such variation was due to the lack of standardisation in the preparation methods and laboratory analysis (Table 3). Variability was also observed intra study with Dai Pre et al. [20] demonstrating that harvesting site could affect cell concentration. In this study, it appeared that lipoaspiration from the thigh resulted in higher cell numbers than the abdomen [20]. This is a key observation when considering the different donor sites across our studies (Table 3). However, more work is required to confirm these findings and establish the best location. Publications have shown other influential factors to be patient demographics [26], harvesting technique [2, 41] and volume processed [68]. The reporting of these factors is variable and has been highlighted in the quality review of studies (Fig. 3). Such non-reproducible results affect the reliability of the concentrations and the subsequent conclusions that can be drawn.

In addition to cell concentration and viability, six studies undertook MSC surface marker analysis to confirm the presence of ASCs within the therapies obtained [12, 16, 17, 27, 65, 66]. The Adiprep system [27] had the highest proportion of MSC CD markers (CD73 60.4%, CD90 65.2%, CD105 33.4%), with Lipocube Nano and Tulip Nanotransfer second and third [17] (CD73 53%, CD90 55.8% and CD73 50%, CD90 42.2% respectively). Despite these results, these studies did not have suitable control methods for comparison (Table 4). Again, these markers only hold particular importance if ASCs are the therapeutic cell type. New information suggests that the other cells within the niche, including: preadipocytes, endothelial cells, macrophages and T-Cells [9, 11], may be just as important (as the ASCs/MSCs act in a paracrine manner). Reporting of these cell subtypes other than just MSCs alone would help us understand the basic science better.

Although these studies have focussed on the cells generated, other authors have highlighted the regenerative capabilities of the cell-free components in processed lipoaspirate. Sarkanen et al. [56] showed that adipogenesis could be induced by using cell-free extract of adipose tissue, possibly due to extracellular vesicles (membrane-bound phospholipids found in the lipoaspirate fluid) [46]. Other factors that could be important include: lipids, RNA, miRNA, DNA, soluble factors and other signalling molecules and proteins, all of which play a role in regulating biological behaviour and immunomodulation [56]. Consideration of using protein assays and other focussed analytical techniques in future studies for these molecules would be useful.

We are still at a juvenile stage in understanding the basic science for these minimally manipulated products, especially given the cellular heterogeneity, small number of ASCs and extracellular components involved. Therefore, improved reporting of their composition is needed so that we can correlate the cellular and molecular components that are present in these therapies with clinical gain [49, 52]. As this review highlights, there is a paucity of studies (four [18, 24, 29, 65]) that have reported not only cellular composition data adequately, but corresponding clinical outcomes as well. Interestingly, these studies were for cosmetic purposes only. The trophic properties of uncultured cells from processed lipoaspirates have been well reported [64], so the use of these POC devices/systems in the aesthetic industry has gained particular traction.

Other publications have reported clinical outcome data alone from using these POC devices/systems (Table 5), but only Lipogems [7, 21, 36, 47, 57–59, 72, 73, 76], MyStem EVO [55] and Hy-Tissue SVF [71] been used in orthopaedic related studies. Lipogems is a closed system which performs washing, filtration and sedimentation, with manual shaking and emulsification also required [74]. It has become popular in orthopaedics, having established an early patent for clinical use [68], as well as being a user-friendly system [68]. Furthermore, its marketing has generated commercial interest amongst consumers. However, as with any marketing, there is the potential for dissemination of false or overexaggerated claims, leading to misunderstanding amongst clinicians [43]. This can hinder further progress within the field. As this review has established, it is not clear what is being reinjected into patients when using these therapies, so it is important that clinicians are made aware of this for their clinical practice.

A weakness of this review is the lack of standardisation in the preparation methods and analytical techniques used across the studies. A systematic review by Robinson et al. [52], which analysed the application of MSCs in orthopaedics and sports medicine, similarly highlighted the inadequate reporting of preparation methods and composition. Standardisation of protocols to allow for fairer comparisons between studies would be helpful. Both the ‘DOSES’ tool [44] and ‘MIBO’ checklist [45] described by Murray et al. were expert consensuses for improving the transparency of cell-based therapy reporting and should be considered in all studies within the field. Another weakness is that some publications may not have been captured if the device/system name was used in the abstract instead of generic search terms (‘device’ or ‘system’). Further studies may have also been missed if they were either unpublished or in non-peer reviewed journals.

## Conclusions

This review increases awareness of POC devices/systems so that users can make informed decisions about using their cellular products for treating musculoskeletal conditions. Regarding cell concentration, cell viability and MSC immunophenotypic analysis, the most effective devices/systems were the manual devices/systems utilising filtration and cutting/mincing techniques. However, it was not known whether high performance in these categories would translate to improved clinical outcomes, let alone which components of the product (cellular or non-cellular) influence the clinical results.

Due to the lack of standardisation in preparation methods and analytical techniques, as well as heterogeneity of the data, it was not possible to draw any reliable conclusions and determine the role of these devices/systems in clinical practice at present. Future studies that investigate clinical outcomes from using these POC devices/systems should improve their reporting of cellular and non-cellular composition (to help to understand the basic science better) as well as pursue minimum standard requirements for preparation protocols and laboratory analysis.

## Supplementary Information

**Additional file 1: Supplementary material.** Search strategy for Medline, EMBASE (combined on Healthcare Databases Advanced Search (HDAS)) and PubMed.

## Data Availability

All data cited and referenced where applicable.

## Abbreviation

OA: Osteoarthritis
MSCs: Mesenchymal stem cells
ASCs: Adipose-derived stem cells
TOST: Total stromal cells
MFAT: Microfragmented adipose tissue
SVF: Stromal vascular fraction
POC: Point-of-care
PRISMA-ScR: Preferred Reporting Items for Systematic Review and Meta-Analysis extension for scoping reviews
MeSH: Medical Subject Heading
RCTs: Randomised control trials
OCEBM: Oxford Centre for Evidence-Based Medicine
MIBO: Minimum Information for Studies Evaluating Biologics in Orthopaedics
STROBE: Strengthening the Reporting of Observational studies in Epidemiology
IFATS: : International Federation for Adipose Therapeutics and Science
