# Supplementary material for: The composition of cell-based therapies obtained from point-of-care devices/systems which mechanically dissociate lipoaspirate: a scoping review of the literature
Source: J Exp Orthop. 2022 Oct 9;9:103. doi: 10.1186/s40634-022-00537-0 (PMC9548462; doi:10.1186/s40634-022-00537-0)
Supplement: Supplementary file 1 — Additional file 1: Supplementary material. Search strategy for Medline, EMBASE (combined on Healthcare Databases Advanced Search (HDAS)) and PubMed. [file 40634_2022_537_MOESM1_ESM.docx]

**The composition of cell-based therapies obtained from point-of-care devices/systems which mechanically dissociate lipoaspirate. A scoping review of the literature.**

**Additional File 1.docx-** Additional Tables 3-5

**Table 3:** Summary of the mechanical devices/systems used in each study, their uncultured cell concentrations, viability (where applicable) and analytical techniques used.

| **Device/ System used**  ***(Author)*** | **Adipose donor site** | **Harvest technique and manipulation of lipoaspirate prior to insertion in device/ system** | **Volume processed (ml)** | **Cell Concentration**  **(x10^6^/ml of lipoaspirate)** | **Cell Viability (%)** | **Estimated total cell yield of product (x10^6^) *** | **Laboratory analysis used to quantify cell numbers (after device/system processing)** | | | | | | |
| --- | --- | --- | --- | --- | --- | --- | --- | --- | --- | --- | --- | --- | --- |
|  |  |  | **Final volume of product (ml)** |  |  |  | **Enzyme use** | **Centrifugation** | **Filtration** | **Washing** | **Other Mechanical** | **Culture medium/ FBS/ Antibiotic** | **Counting Device** |
| Adinizer  *(Copcu et al*[18]*)* | Abdomen | Harvested with 2.8mm diameter cannula with tumescent solution and adrenaline. Predilution with saline in 50% of samples tested | 5-20 | 1.22** | 92.75** | 1.13- 13.6 (Depending on volume used) |  | Y |  |  |  |  | LunaStem device |
|  |  |  | 1-12  (Variable) |  |  |  |  |  |  |  |  |  |  |
| Adiprep  *(Dragoo et al*[27]*)* | Knee fat pad | Harvested during arthroscopy into AquaVage system. Then subjected to fractionisation and syringe emulsification. | 30 | 0.486*** | 69.03*** | 0.99  (Mean) | Y | Y |  | Y |  | Y | Haemocytometer |
|  |  |  | ~2.95  (Mean) |  |  |  |  |  |  |  |  |  |  |
| Fastem  *(Domenis et al*[24]*)* | Abdomen, hips and trochanter region | Harvesting procedure not mentioned. ‘Standardised procedural protocol’ not described. | No data | 0.444 to 1**** | - | N/A | Y | Y | Y |  |  |  | No data |
| Fastem and MyStem  *(Gentile et al*[29]*)* | No data | Harvesting procedure not mentioned. | 80 | 0.03 and 0.005 | 98***** | 0.29 and 0.049 |  | Y | Y |  |  | Y | Haemocytometer |
|  |  |  | 10 |  |  |  |  |  |  |  |  |  |  |
| Hy-Tissue SVF  *(Busato et al*[12]*)* | Abdomen | Harvested with 11G cannula with Klein tumescence solution, followed by decantation | 25-30 | 0.041 | - | N/A |  | Y |  |  |  | Y | CytoSMART counter |
|  |  |  | No data |  |  |  |  |  |  |  |  |  |  |
| Lipocube Nano &  Tulip Nanotransfer  *(Cohen et al*[17]*)* | No data | Harvested with 2.4mm diameter cannula and then cleaned with Ringer’s lactate, sedimented and decanted. | 10 | 2.24 and 1.44 | 96.05 | N/A | Y | Y |  |  | Y |  | Muse Flow Cytometer |
|  |  |  | No data  (‘Pellet’ used) |  |  |  |  |  |  |  |  |  |  |
| Lipocube SVF  *(Tiryaki et al*[66]*)* | Hip | Harvested with 3.5mm diameter cannula then decanted. | 20 | 0.94 | 97.55 | N/A |  |  |  |  | Y | Y | MuseCell Analyzer |
|  |  |  | No data  (‘Pellet’ used) |  |  |  |  |  |  |  |  |  |  |
| Lipogems  *(Vezzani et al*[75]*)* | Abdomen | Harvested with 17G cannula either manually or vacuum assisted and mixed with saline | 60 | 0.027 | - | N/A | Y | Y | Y | Y |  | Y | Haemocytometer |
|  |  |  | 20-30 |  |  |  |  |  |  |  |  |  |  |
| Lull pgm  *(Morselli et al*[42]*)* | Abdomen | Harvesting procedure not mentioned. ‘Negative pressure’- not clarified. | 30 | 2.4 | - | N/A | Y | Y | Y |  | Y | Y | Cell Coulter counter |
|  |  |  | 10 |  |  |  |  |  |  |  |  |  |  |
| MyStem  *(Cicione et al*[16]*)* | No data | Harvested with MyStem 1.8mm blunt- tip cannula. Process not reported. | 17-50 | 0.6 | 75.87 | 3.6- 10.7 (Depending on introduced volume) |  | Y |  |  |  |  | NucleoCounter |
|  |  |  | 8-23.5  (Variable) |  |  |  |  |  |  |  |  |  |  |
| MyStem  *(Tarallo et al*[65]*)* | Abdomen | Harvested with local anaesthetic. ‘Standard protocol’- not described | 30 | 0.83 | 74.3 | 0.62- 4.3 | Y | Y |  |  |  | Y | NucleoCounter |
|  |  |  | 1-7  (Variable) |  |  |  |  |  |  |  |  |  |  |
| Puregraft  *(Streit et al*[63]*)* | Abdomen | Harvested with 3.5mm diameter cannula with tumescent solution. | 50 | 0.198 | 60 | N/A | Y | Y |  | Y |  | Y | Haemocytometer |
|  |  |  | No data  (Pellet used) |  |  |  |  |  |  |  |  |  |  |
| Rigenera  *(Dai Pre et al*[20]*)* | Thigh and Abdomen | Harvesting procedure not mentioned. Lipoaspirate mixed with equal volume of culture medium, FBS and antibiotics. | 4 | 21 | - | N/A |  | Y | Y |  |  | Y | Tryptan blue exclusion assay |
|  |  |  | 4 |  |  |  |  |  |  |  |  |  |  |
| Transpose RT  *(Winnier et al*[77]*)* | No data | Harvested with ‘standard procedure’- not described. Lipoaspirate mixed with lactated Ringer solution | 25 | 0.084 | 61.7 | 0.16 |  |  |  |  |  |  | NucleoCounter |
|  |  |  | 3 |  |  |  |  |  |  |  |  |  |  |
| Tulip Nanotransfer  *(Sese et al*[61]*)* | Abdomen | Harvested with Carraway Harvester cannula with tumescent fluid, then washed with saline. | 20 | 6.63 | 76.8 | 50.9 |  | Y |  |  |  | Y | NucleoCounter |
|  |  |  | 10 |  |  |  |  |  |  |  |  |  |  |

*Estimated total cell yield= Volume of product (ml) X Cell concentration (x10^6^/ml of lipoaspirate) X % Cell viability

**Value given is an average obtained from the four different protocols used in the study

***Figures from Layer 2 which resulted in the highest numbers

****Enrichment performed in only 50% of lipoaspirate sample

*****Generalised figure for the study overall, not specific to either device/system

**Table 4:** Immuno-phenotypic analysis performed and CD Marker Expression.

| **Device/ System used**  ***(Author)*** | **Type of immuno-phenotypic analysis of cell subtypes** | **Terminology for uncultured, freshly isolated cells** | **Stage of cell processing** | **Positive cell CD marker expression (%)** | | | | | | | | | | | |
| --- | --- | --- | --- | --- | --- | --- | --- | --- | --- | --- | --- | --- | --- | --- | --- |
|  |  |  |  | **Mesenchymal stem cell markers**  **CD markers observed in Pericytes as well* | | | | | | | **Endothelial cell, pericyte and haematopoetic markers** | | | | |
|  |  |  |  | **CD 13** | **CD 29** | **CD 44*** | **CD 73** | **CD 90*** | **CD 105*** | **CD 146*** | **CD 31** | **CD 34** | **CD 45** | **CD 68** | **Other** |
| Adinizer  *(Copcu et al*[18]*)* | Flow Cytometry | Stromal cells/ Nuclear cells | Immediately after device use (minimally manipulated) | As per methods- Proportions of CD45 negative cells were analysed in CD34−CD146+ and CD34+CD146−CD90+ (deemed as regenerative perivascular cells), and CD34+CD146+ as endothelial cells. However, percentages not specifically reported in results. | | | | | | | | | | | |
|  |  |  | No control |  |  |  |  |  |  |  |  |  |  |  |  |
|  |  |  | Passage in culture following device (extensively manipulated) |  |  |  |  |  |  |  |  |  |  |  |  |
| Adiprep  *(Dragoo et al*[27]*)* | Flow Cytometry | SVF Cells | Immediately after device use (minimally manipulated) | 56.5 |  | 72.0 | 60.4 | 65.2 | 33.4 |  |  |  | 80.3 |  |  |
|  |  |  | No control |  |  |  |  |  |  |  |  |  |  |  |  |
|  |  |  | Passage in culture following device (extensively manipulated) | 94.3 |  | 96.6 | 97.0 |  |  |  |  |  |  |  |  |
| Fastem  *(Domenis et al*[24]*)* | Flow Cytometry | SVF Cells | Immediately after device use (minimally manipulated) |  |  |  |  |  |  |  |  |  | 50-60 |  | **CD34+CD45-CD31-**  10-20 |
|  |  |  | Control- ‘modified’ Coleman’s procedure (centrifugation) |  |  |  |  |  |  |  |  |  | 0-10 |  | **CD34+CD45-CD31-**  20-30 |
|  |  |  | Passage in culture following device (extensively manipulated) |  |  |  |  |  |  |  |  |  |  |  |  |
| Fastem and MyStem  *(Gentile et al*[29]*)* | Not done | SVF Nucleated Cells |  |  |  |  |  |  |  |  |  |  |  |  |  |
| Hy-Tissue SVF  *(Busato et al*[12]*)* | Flow Cytometry | Free nucleated SVF cells | Immediately after device use (minimally manipulated) |  |  |  | 7.61 |  | 6.28 | 2.6 |  | 9.91 | 5.5 | 3.5 | **CD116** 0.7 |
|  |  |  | Control- enzymatic digestion using 0.1% collagenase type I at 37 °C for 45min followed by centrifugation at 400G for 10min. |  |  |  | 10.1 |  | 9.98 |  |  | 3.67 |  |  |  |
|  |  |  | Passage in culture following device (extensively manipulated) |  | 90 | >90 | >70 | 60 | 90 |  |  |  |  |  |  |
| Lipocube Nano  *(Cohen et al*[17]*)* | Flow Cytometry | SVF Cells | Immediately after device use (minimally manipulated) | 42.0 |  |  | 53 | 55.8 |  | 53.2 |  | 18.8 |  |  |  |
|  |  |  | No control |  |  |  |  |  |  |  |  |  |  |  |  |
|  |  |  | Passage in culture following device (extensively manipulated) |  |  |  |  |  |  |  |  |  |  |  |  |
| Lipocube SVF  *(Tiryaki et al*[66]*)* | Flow Cytometry | Nucleated SVF Cells | Immediately after device use (minimally manipulated) |  |  | 21.5 | 6.16 | 11.4 | 9.0 |  |  |  |  |  |  |
|  |  |  | Control- enzymatic digestion using GMP grade collagenase NB6 at a concentration of 0.1 U/ml at 37 °C for 30min followed by centrifugation at 400G for 10min. Then washed with PBS solution and centrifuged at 300G for 5min. |  |  | 6.93 | 3.44 | 5.88 | 3.06 |  |  |  |  |  |  |
|  |  |  | Passage in culture following device (extensively manipulated) |  |  |  |  |  |  |  |  |  |  |  |  |
| Lipogems  *(Vezzani et al*[75]*)* | Flow Cytometry | SVF Nucleated Cells | Immediately after device use (minimally manipulated) |  |  |  |  |  |  |  |  |  |  |  | **CD146+CD34** 33.5  **CD34+CD146** 5.46 |
|  |  |  | No control |  |  |  |  |  |  |  |  |  |  |  | **CD146+CD34** 8.39  **CD34+CD146** 51.5 |
|  |  |  | Passage in culture following device (extensively manipulated) | CD14, CD31, CD40 ligand (CD154) significantly more abundant than when compared to control. | | | | | | | | | | | |
| Lull pgm  *(Morselli et al*[42]*)* | Not done | SVF Cells |  |  |  |  |  |  |  |  |  |  |  |  |  |
| MyStem  *(Cicione et al*[16]*)* | Flow Cytometry | Lipoaspirate fluid cells | Immediately after device use (minimally manipulated) |  |  | <0.1 |  | 1-1.5 | <0.1 |  | 0.5-1 | <0.5 | <1 |  |  |
|  |  |  | Control- centrifugation ‘as previously described’ |  |  | <0.1 |  | 1.5-2 | <0.1 |  | 1 | <0.5 | <0.5 |  |  |
|  |  |  | Passage in culture following device (extensively manipulated) |  |  | 93 | 98 | 95 | 96 |  |  |  |  |  |  |
| MyStem  *(Tarallo et al*[65]*)* | Flow Cytometry | Freshly isolated LAF Cells | Immediately after device use (minimally manipulated) |  |  |  | 0-10 | 75 | 0-10 |  |  | 0-10 | 20 |  | **CD31** 30 |
|  |  |  | No control |  |  |  |  |  |  |  |  |  |  |  |  |
|  |  |  | Passage in culture following device (extensively manipulated) | All culture-expanded cells displayed an ASC-like immunophenotype: CD105+, CD73+, CD90+, CD45- and CD34-CD31. | | | | | | | | | | | |
| Puregraft  *(Streit et al*[63]*)* | Direct Immunofluorescence | SVF Cells | Immediately after device use (minimally manipulated) | Analysed adhesive properties to determine stem cell nature.  All adherent cells were positive for CD90 and CD105 and negative for CD31 and CD45 antigens (stem cell marker). Numbers not specified. | | | | | | | | | | | |
|  |  |  | Control 1- aliquot was left at 37°C  for 20min under the action of gravity (decantation).  Control 2- aliquot centrifuged at 1200G for 3 min. |  |  |  |  |  |  |  |  |  |  |  |  |
|  |  |  | Passage in culture following device (extensively manipulated) |  |  |  |  |  |  |  |  |  |  |  |  |
| Rigenera  *(Dai Pre et al*[20]*)* | Flow Cytometry | Total cells | Immediately after device use (minimally manipulated) |  |  |  |  |  |  |  |  | 3.12 | 4.98 |  | **CD44/CD90** 30.4  **CD73/CD105** 16.6  **CD73/29** 27.8 |
|  |  |  | Control- enzymatic digestion using 0.1% collagenase type I at 37 °C for 45min in Hank’s Balanced Salt Solution (HBSS) and 2% bovine serum albumin followed by centrifugation at 3000 rpm for 7min. |  |  |  |  |  |  |  |  | 76.7 | 7.32 |  | **CD44/CD90** 48.1  **CD73/CD105** 54.3  **CD73/29** 62 |
|  |  |  | Passage in culture following device (extensively manipulated) | Expression of the typical mesenchymal stem cell markers (CD105, CD90, CD73, CD44, and CD29) and the hematopoietic markers (CD45 and CD34) was preserved through culture passages. | | | | | | | | | | | |
| Transpose RT  *(Winnier et al*[77]*)* | Not done | Adipose-derived regenerative cells |  | | | | | | | | | | | | |
| Tulip Nanotransfer  *(Cohen et al*[17]*)* | Flow Cytometry | SVF Cells | Immediately after device use (minimally manipulated) | 18.3 |  |  | 50 | 42.1 |  | 24.1 |  | 7.9 |  |  |  |
|  |  |  | No control |  |  |  |  |  |  |  |  |  |  |  |  |
|  |  |  | Passage in culture following device (extensively manipulated) |  |  |  |  |  |  |  |  |  |  |  |  |
| Tulip Nanotransfer  *(Sese et al*[61]*)* | Not done | Nanofat cells |  |  |  |  |  |  |  |  |  |  |  |  |  |

**Table 5:** Device/system characteristics and clinical applications in literature.

| **Device/ System** | **Company and location** | **Level of automation** | **Processing Time (mins)** | **Mechanical techniques used by device/system** | | | | | | **Clinical Applications in PubMed indexed studies** |
| --- | --- | --- | --- | --- | --- | --- | --- | --- | --- | --- |
|  |  |  |  | Centrifugation | Filtration | Cutting / Mincing | Sedimentation/ Decantation | Washing | Other (Specify) |  |
| Adinizer | BSLrest, Busan, South Korea | Manual | Variable (operator dependent) |  |  | Y |  |  |  | **Indication:** Fat grafting/ Lipofilling  **Treatment**[18]**:** Cellular product applied at varying depths to different aesthetic units of the face in 24 patients.  **Outcome:** Visual analog scale (VAS) scores at 2 years were consistently high (Range 6-9) from both patient and surgeon. |
| Adiprep system (+ Smartprep) | Harvest Technologies Corp. Plymouth, MA, USA | Manual + Automated | 4 | Y |  |  |  |  | Emulsification | None |
| Fastem | CORIOS Soc. Coop, Milan, Italy | Automated | 10 | Y | Y |  |  |  |  | **Indication:** Fat grafting  **Treatment**[24]**:** Cellular product used to enrich fat grafts before breast augmentation in six patients, comparing their clinical results with patients who underwent grafting with standard lipoaspirate (n=16).  **Outcome:** Greater gain of thickness of both the central and superior-medial quadrants at 6 months vs control. |
| Hy- Tissue SVF | Fidia Farmaceutici S.p.A, Padua, Italy | Manual | 15 |  | Y |  | Y |  | Massage | **Indication:** Osteoarthritis (Animal in vitro study)[22]  **Indication:** Achilles tendinopathy  **Treatment**[71]: 21 patients with non-insertional achilles tendinopathy (28 tendons) were treated unilaterally or bilaterally with autologous cellular product.  **Outcome:** Significant improvements in VAS, AOFAS and VISA-A scores at 15 and 30 day follow up intervals vs PRP group. |
| Lipocube Nano | Lipocube Inc, London, UK | Manual | 20-30 |  | Y | Y |  |  | Emulsification | None |
| Lipocube SVF/ CellDrive | Lipocube Inc, London, UK | Manual + Automated | 20-30 | Y | Y | Y |  |  |  | **Indication:** Fat grafting  **Treatment**[67]**:** SVF cell-enriched fat grafting in 46 patients for various aesthetic and reconstructive applications.  **Outcome:** No complications. Results on a 4-point patient satisfaction scale ranged from good to excellent. |
| Lipogems | Lipogems International S.p.A, Milan, Italy | Semi- automated | 3-5 |  | Y |  | Y | Y | Shaking, Emulsification | **ENT**  **Indication:** Vocal cord palsy  **Treatment**[54]**:** 3 patients had laryngoplasty and injection of autologous cellular product.  **Outcome:** At 12-month follow-up period, voice improvement was consistent in all patients.  **General Surgery**  **Indication:** Intersphincteric anal lipofilling  **Treatment**[15]: 3 patients with faecal incontinence had autologous cellular product injected in the intersphincteric anal groove.  **Outcome:** At 1 month post procedure, each patient had an improved Wexner incontinence score. At 6 months, ano-rectal manometry showed an increase of resting pressure and ultrasound showed increased thickness of the sphincter.  **Indication:** Repair of a vesicouterine fistula  **Treatment**[62]: 1 patient had endoscopic transurethral resection of the fistulous tract and injection of autologous cellular product.  **Outcome:** 3 months post procedure, patient was asymptomatic. Cystoscopy showed appropriate scar tissue and cystogram revealed complete repair of VUF. At 24 months, there were no recurrences.  **Orthopaedic Surgery**  **Indication:** Osteoarthritis  **Treatment**[7]: 20 patients with knee OA were injected with autologous cellular product and followed up at various intervals.  **Outcome:** Improvements in Knee injury and Osteoarthritis Outcome Score (KOOS) were significant at 3-,6- and 12-months follow-up. At one year, there were improvements in KOOS pain= 14 points, symptoms= 7, activities of daily living= 13, sports= 19 and quality of life=15.  **Treatment**[76]: 25 patients with shoulder OA were injected with autologous cellular product and followed up at various intervals.  **Outcome:** At one-year, significant improvement (p<0.001) in Visual Analog Scale and disabilities of the arm.  **Treatment**[21]: 6 consecutive patients with hip OA were given single intra-articular injection of autologous cellular product and followed up at 6 months.  **Outcome:** Harris Hip Score improved from 67.2 (mean pre-operative value) to 84.6 (mean pre- post-operative value)  **Treatment**[47]: 17 patients with knee OA treated with ultrasound-guided intra-articular injection of autologous cellular product and followed up for up to 12 months.  **Outcome:** Knee Society Score improved from average 74 (baseline) to 82 (12 months)  **Treatment**[59]: 20 patients with temporomandibular OA treated with autologous cellular product after arthrocentesis vs control group (hyaluronic acid instead). Follow up for up to 6 months.  **Outcome**: Treatment group had a statistically significant superiority in the success rate compared with the control group (P = .018).  **Treatment**[72]: 64 patients with symptomatic mild-severe knee OA treated with autologous cellular product. Follow up for up to 12 months.  **Outcome**: KOOS, NRS and EQ-5D improved significantly at follow-up compared to baseline (p < 0.05).  **Treatment**[57]: 52 patients with early knee OA treated with autologous cellular product after arthroscopic debridement. Follow up for up to 24 months.  **Outcome**: The IKS function score improved from average 57.2 (pre-operatively) to 83.0 (at the latest follow-up) (p<0.01).  **Treatment**[73]: 23 patients with early to moderate patellofemoral OA treated with autologous cellular product. Mean follow-up was 22.1 months.  **Outcome**: Significant improvements in mean IKS knee and function scores vs baseline (35.6 to 61.9 and 52.0 to 82.3 respectively).  **Treatment**[58]**:** 202 patients with OA (Kellgren-Lawrence I-IV) were injected with autologous cellular product. Mean follow-up was 24.5 months.  **Outcome:** At 6 months, Total KOOS significantly improved from baseline (p 0.001) and between 6-12 months. At 6 months, VAS was reduced vs baseline (p 0.001), increased at 12 months but remained below baseline.  **Indication:** Used with High Tibial Osteotomy (HTO) for correction of varus knee OA  **Treatment**[36]: 42 patients treated with HTO and simultaneous intra articular injection of cellular product vs 43 patients treated with only HTO.  **Outcome:** No significant results between both treatment groups in terms of KOOS pain, symptoms, sports, and quality of life. However, a significant improvement (p<0.05) in the activities of daily living. |
|  |  |  |  |  |  |  |  |  |  |  |
| Lull pgm system | *Not patented by company* | Manual | Variable |  |  |  | Y | Y |  | None |
| MyStem | MyStem LLC, Wilmington, DE, USA | Automated | 5-10 |  | Y |  |  | Y |  | **Indication:** Osteoarthritis  **Treatment**[55]: 84 consecutive patients with knee OA treated with intra-articular injection of autologous cellular product. Follow up at 12 months.  **Outcome**: There were significant improvement in pain scores (NRS decrease by 3.5 (p<0.001), WOMAC pain by 7.02 (p<0.001), WOMAC stiffness by 1.97 (p<0.001).  **Indication:** Breast reconstruction  **Treatment**[29]: Cellular product used to treat breast soft-tissue defects in 10 patients (10 for each device/system).  **Outcome:** There was 43% maintenance of contour restoration after 1 year, respectively.  **Indication:** Treatment of anal fistula  **Treatment**[35]: 1 patient with a left posterior lateral perianal abscess and fistula tract, injected with autologous cellular product.  **Outcome:** Anoscopy and transanal ultrasound showed that the fistula had completely healed.  **Indication:** Leg ulcers  **Treatment**[33]: 31 patients who had non healing venous ulcers and had responded to previous venous surgery were treated with autologous cellular product.  **Outcome:** At 12 month follow up, 18 had completely healed and the other 13 showed contraction and epithelialisation.  **Indication:** Wound healing  **Treatment**[65]**:** Cellular product applied to wounds in 20 patients, comparing their results against an equal cohort who used a conservative approach.  **Outcome:** Treatment group achieved a slightly faster healing time (22.3 days versus 24.9 days, p<0.05) and greater aesthetic results (p<0.05). |
| Puregraft | Cytori Therapeutics. Inc, San Diego, California, USA | Manual | 5-6 |  | Y |  | Y | Y | Squeezing | **Indication:** Fat grafting  **Treatment**[38]- 15 patients underwent breast reconstruction with autologous cellular product.  **Outcome:** Significant improvement in BREAST-Q scores post-operatively compared to pre-operative scores.  **Indication:** Treatment of alopecia  **Treatment**[34]: 22 patients with varying hairloss had autologous cellular product (Puregraft fat and 0.5 × 10^6 ASCs) injected into skin and subcutaneous layer of the scalp.  **Outcome:** No adverse events. Significant increase in terminal hair count at one year follow up for Norwood-Hamilton 3 cohort (men with early hair loss). |
| Rigenera | Rigenera- HBW, Turin, Italy | Automated | 3-4 |  | Y | Y |  |  |  | **Indication:** Treatment of chronic ulcers  **Treatment (using skin biopsy)**[39]: 15 patients with chronic ulcers and treated with micrograft (cellular product and scaffold of equine collagen).  **Outcome:** 86.7% ulcers were healed at 16 weeks post procedure and the results maintained at 6 months.  **Indication:** Skin grafting  **Treatment (using skin biopsy)**[51]: 70 patients with traumatic wounds of the lower and upper limbs, treated with cellular product.  **Outcome:** Complete healing of wounds in 69 cases at 35 to 84 days post procedure.  **Indication:** Wound closure  **Treatment (using skin biopsy)**[37]: 3 patients with wound dehiscence following orthopaedic surgical interventions treated with cellular product.  **Outcome:** Remission of wound dehiscence at 15 to 60 days post procedure. |
| Transpose RT | InGeneron. Inc, Houston, TX, USA | Semi- automated | 45-55 | Y | Y |  |  | Y | Acceleration + deceleration | None |
| Tulip NanoTransfer | Tulip Medical, San Diego, CA, USA | Manual | Variable |  | Y | Y |  |  | Emulsification | None |
